# Tunable Filters Using Defected Ground Structures at Millimeter-Wave Frequencies

**DOI:** 10.3390/mi16010060

**Published:** 2024-12-31

**Authors:** Kaushik Annam, Birhanu Alemayehu, Eunsung Shin, Guru Subramanyam

**Affiliations:** Center of Excellence for Thin-Film Research and Surface Engineering (CETRASE), Department of Electrical and Computer Engineering, University of Dayton, Dayton, OH 45469, USA; biredesta@gmail.com (B.A.); eshin1@udayton.edu (E.S.); gsubramanyam1@udayton.edu (G.S.)

**Keywords:** defected ground structure (DGS), phase change material (PCM), frequency tuning, dumbbell u-slot, reconfigurable devices, tunable filters, BST tunable filters, tunable band stop filters, GeTe tunable filters, high-power filters

## Abstract

This paper explores the potential of phase change materials (PCM) for dynamically tuning the frequency response of a dumbbell u-slot defected ground structure (DGS)-based band stop filter. The DGSs are designed using co-planar waveguide (CPW) line structure on top of a barium strontium titanate (Ba_0.6_Sr_0.4_TiO_3_) (BST) thin film. BST film is used as the high-dielectric material for the planar DGS. Lower insertion loss of less than −2 dB below the lower cutoff frequency, and enhanced band-rejection with notch depth of −39.64 dB at 27.75 GHz is achieved by cascading two-unit cells, compared to −12.26 dB rejection with a single-unit cell using BST thin film only. Further tunability is achieved by using a germanium telluride (GeTe) PCM layer. The electrical properties of PCM can be reversibly altered by transitioning between amorphous and crystalline phases. We demonstrate that incorporating a PCM layer into a DGS device allows for significant tuning of the resonance frequency: a shift in resonance frequency from 30.75 GHz to 33 GHz with a frequency shift of 2.25 GHz is achieved, i.e., 7.32% tuning is shown with a single DGS cell. Furthermore, by cascading two DGS cells with PCM, an even wider tuning range is achievable: a shift in resonance frequency from 27 GHz to 30.25 GHz with a frequency shift of 3.25 GHz is achieved, i.e., 12.04% tuning is shown by cascading two DGS cells. The results are validated through simulations and measurements, showcasing excellent agreement.

## 1. Introduction

Prior to 5G, the sub-3 GHz band, i.e., LTE bands were used for mobile communications. However, due to increasing demand for data-intensive applications like IoT, autonomous vehicles and other emerging technologies, 5G has evolved. The 5G network technology is intended to address these demands by establishing communication data links in the millimeter-wave (mmW) spectrum. The mmW frequency band of 5G is still unexplored due to lack of commercial technology and complex design challenges, but it may soon be feasible to rapidly increase data rates utilizing sub-6 GHz and mmW frequency bands using carrier aggregation (CA) techniques. The CA technique combines multiple bands to achieve wider bandwidth, allowing higher data rates. Currently, Wi-Fi 6E/7 offers 160 MHz and 320 MHz channels. The number of bands that can be carrier-aggregated has increased with the inception of 5G compared to 4G long-term evolution (LTE). This results in a greater number of filters needed in a single module, which significantly intensifies the challenges associated with filter design.

Filters continue to play a crucial role in the smooth operation of the RF (radio frequency) system, especially at mmW frequencies where noise is predominant. Several researchers have demonstrated band stop filter (BSF) [1,2,3,4] and band pass filter (BPF) [5,6] on-chip passive circuits to provide a system-on-a-chip (SOC) solution and stringent process control at mmW frequencies. As small variations in material thickness could lead to larger shifts in the performance specifications at these frequencies, the authors of [1,2,3,5,6] use complementary metal oxide semiconductor (CMOS) technology, while [4] uses Bi-CMOS technology. However, the main disadvantage of on-chip passive circuits is their low Q-factor along with increased cost due to larger chip area. Moreover, microelectronic fabrication and packaging techniques have evolved significantly, allowing precision material tolerances which makes it feasible to integrate off-chip solutions efficiently. Bulk acoustic wave (BAW) resonator filters have proven to be a reliable solution to meet stringent specifications up to 7 GHz frequencies. Several groups are working on acoustic resonator filters for mmW frequency bands; in [7], a periodically polarized piezoelectric film (P3F) bulk acoustic wave resonator using an aluminum scandium nitride (AlScN) piezoelectric thin film with scandium (Sc) doping concentration of 20% and 30% operating in overtone mode is presented, where the coupling factor significantly reduces for the higher order modes. The authors of [8] also present a P3F bulk acoustic resonator at 19 GHz operating in TE3 mode where electrode thickness has been increased to improve Q, which shifts the series resonance frequency down as the overall thickness of the resonator is increased, which in turn reduces the coupling factor. In [9], 28 GHz resonators using aluminum nitride (AlN) thin films are presented which have higher losses. In general, acoustic filters at mmW frequency bands suffer from reduced coupling and large impedances which make them difficult to match to 50 Ω, and fabrication becomes tedious as the resonator sizes become too small, resulting in low power failures. However, research is still underway to overcome these challenges for implementation of bulk acoustic filters for mmW frequencies. In [10], the authors presented a 7^th^-order low pass filter (LPF) at 28 GHz and 5^th^-order BPF for 28 GHz and 39 GHz frequency bands using epoxy film from Aginomoto (ABF GL102); however, the device dimensions are large.

BSFs are significant in rejecting unwanted signals as they selectively attenuate a specific range of frequencies reducing noise interference and improving signal-to-noise ratio.

The number of filters required in an RF front end module has been increasing rapidly. This increases the design complexity of the module as size is a big constraint for many applications. Having a tunable filter would reduce the number of filters that need to be integrated into the system.

In [11], MEMS-based tunable BSF and BPF have been demonstrated for millimeter wave applications. The study uses two cantilever shunt switches to achieve tunable behavior in the 60 GHz band. In this paper, a novel tunable filter technique is demonstrated, by integrating two different materials technologies. A defected ground structure-based tunable mmW band stop filter using barium strontium titanate (BST) and germanium telluride (GeTe) thin films is presented. This reconfigurable filter is feasible to implement and possesses advantages over traditional micromachining techniques. Moreover, thin films can handle higher power which makes it more suitable for high power applications.

A defected ground structure is similar to a photonic band gap (PBG) structure, featuring defects etched within the ground planes. These etched defects disrupt the shield current distributions resulting in an increase of inductance and capacitance of the transmission line [12]. The distribution of the shielded currents is determined by the geometry and dimensions of the defects, while the band gap property relies on multiple design factors such as lattice shape, lattice spacing and the number of lattices [13]. Ahn et al. [13] have proposed DGSs by introducing a thin slot in between two square-shaped photonic band gap cells.

## 2. Materials and Methods

### 2.1. Materials

Barium strontium titanate (Ba_0.6_Sr_0.4_TiO_3_) and germanium telluride (Ge_0.5_Te_0.5_) targets were purchased from Kurt J. Lesker Co., Jefferson Hills, PA, USA. C-plane sapphire substrates (Al_2_O_3_) were obtained from Nova Electronic Materials Inc., Flower Mound, TX, USA. All raw chemical reagents were of analytical grade and used without further purification.

### 2.2. Method

#### Design and Fabrication of DGS Devices

A co-planar waveguide (CPW) line configuration-based DGS filter is presented in this paper. This design features an integrated approach by combination of a conventional dumbbell DGS and u-slot to achieve lower resonance frequency with smaller defect sizes, sharper cutoff and lower insertion loss. The dumbbell u-slot design used in this work was first proposed by our team [14,15] using microstrip transmission line configuration. Here, it is redesigned to a CPW configuration as it has better advantages in terms of integration with other circuits and packaging. DGSs can operate effectively over a wide range of frequencies, providing designers with an extra degree of freedom. A significant benefit of DGSs over PBG structures is the circuit area. DGSs can achieve similar performance parameters compared to periodic parameters while also exhibiting slow-wave effects.

**Figure 1:**
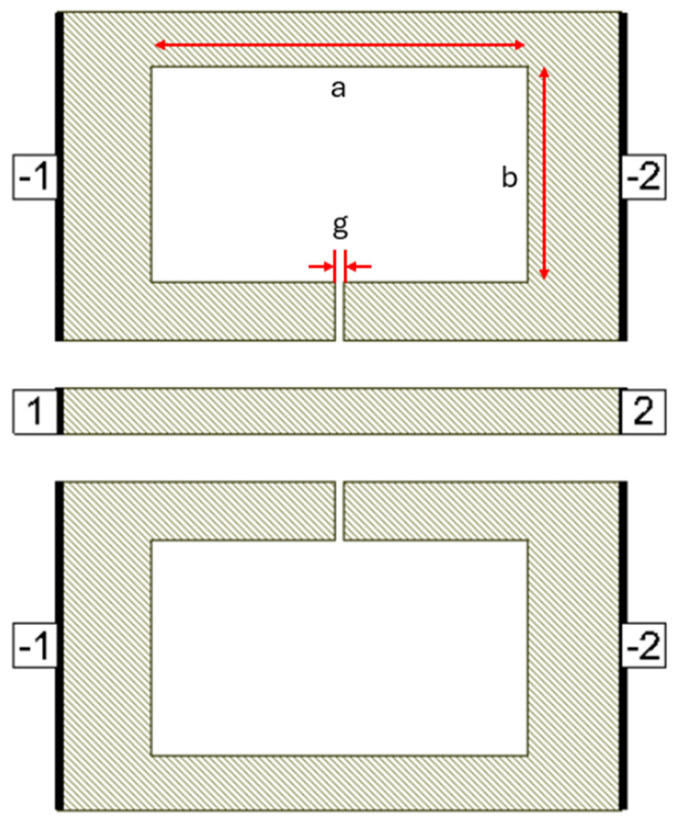
Dumbbell DGS on CPW line.

**Figure 2:**
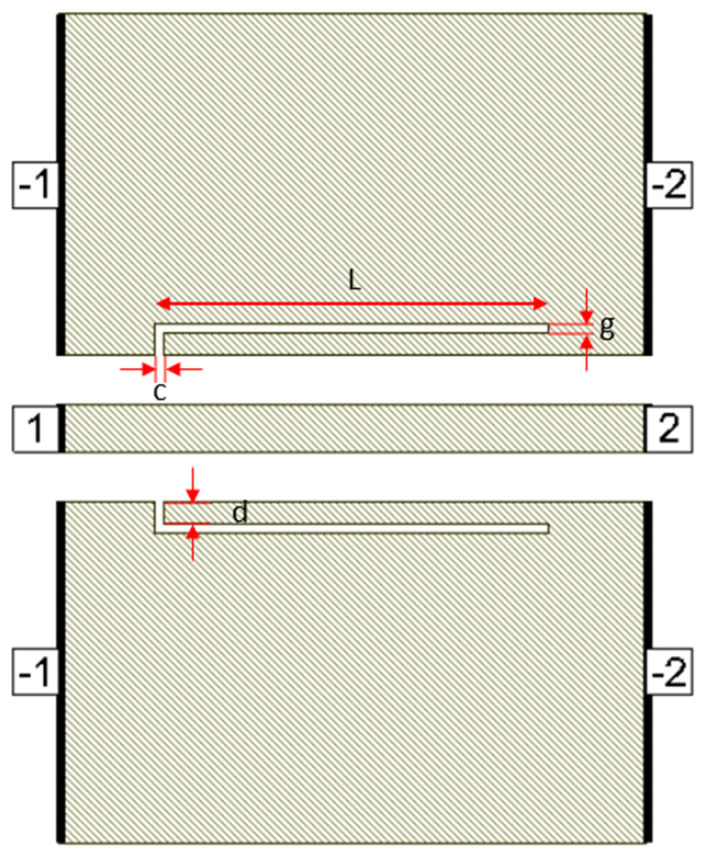
U-slot DGS on CPW line.

Defects in the ground plane of transmission lines can have different geometrical shapes, resulting in determining transmission line characteristics and yielding different frequency responses. Many researchers have proposed different defect shapes [13,14,16,17,18,19] for sub-6 GHz frequency band and [3] for mmW frequency band. The dumbbell DGS shown in Figure 1, introduced in [13], using microstrip transmission line has narrow and wide etched areas. These defects increase the effective inductance and capacitance of the transmission line. The rectangular patch a × b has dimensions of 400 µm × 230 µm and the gap width g is 10 µm. The u-slot DGS shown in Figure 2, introduced in [16], also uses a microstrip line and is redesigned for CPW line configuration. Slot length L is 402.5 µm, distance d is 22.5 µm and slot width c = g = 10 µm.

**Figure 3:**
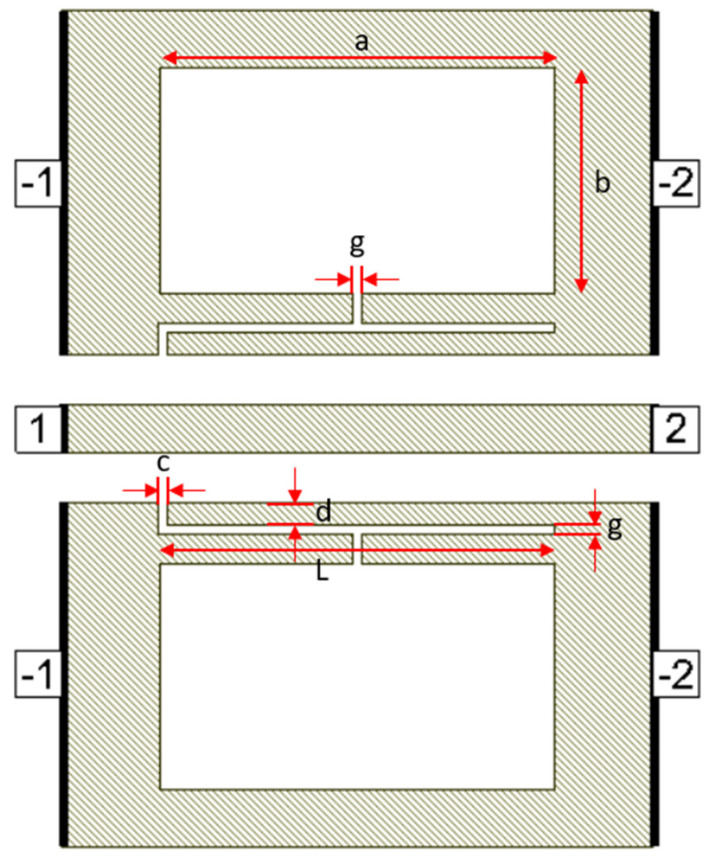
Dumbbell u-slot DGS on CPW line.

Figure 3 shows a dumbbell u-slot DGS structure using CPW line configuration. In this design, both dumbbell and u-slot DGS are integrated. The u-slot has more capacitance as the thin slot forms a capacitor, being two metal surfaces with a dielectric in between. The dumbbell shape has more inductance with less capacitance, as the rectangular patch makes the current pass through a longer path, thus introducing inductor-like behavior. By integrating both, we can achieve more capacitance and inductance with the same size defects. This improves the filter performance, and a lower resonance frequency is achieved. Defects are symmetrical along the line of signal, i.e., both the ground planes are symmetrical. The CPW line is designed to have a characteristic impedance of 50 Ω.

**Figure 4:**
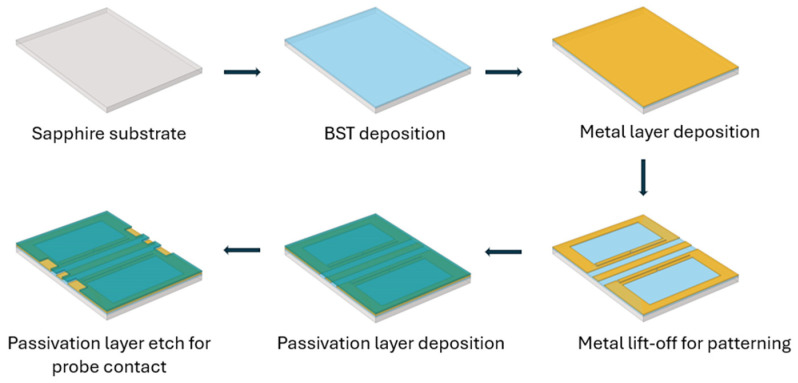
Schematic representation of the fabrication process of a dumbbell u-slot DGS using CPW line configuration with BST thin film.

The designed DGS devices were fabricated on a C-cut sapphire substrate, as shown in Figure 4. BST thin films are deposited on the wafer using the pulsed laser deposition (PLD) technique. The substrate is heated to 900 °C before deposition and kept at this temperature during deposition. Oxygen gas is then released and is maintained at 75 mTorr partial pressure during deposition. A beam of high-power laser pulses using a KrF excimer laser operating at 248 nm wavelength with a pulse repetition frequency of 30 Hz and laser energy about 175–180 mJ is directed towards a BST target to deposit a 500 nm thin film of BST on the surface of the wafer [20,21]. On top of the BST, a 500 nm thick Ti/Pt/Au (20/100/380 nm) metal composition is deposited. A thin layer of titanium (Ti) is used for adhesion, followed by a platinum (Pt) thin film which acts as the oxygen-permeable layer, and finally gold (Au) to create thicker metal contact. DGSs were created using this metal composition. These metals are deposited using the e-beam evaporation technique. The lift-off process is used to remove metal to create defects in the ground plane. Finally, a 250 nm silicon nitride (Si_3_N_4_) thin film is deposited for passivation and etched for probe contacts, as seen in Figure 4.

## 3. Results

**Figure 5:**
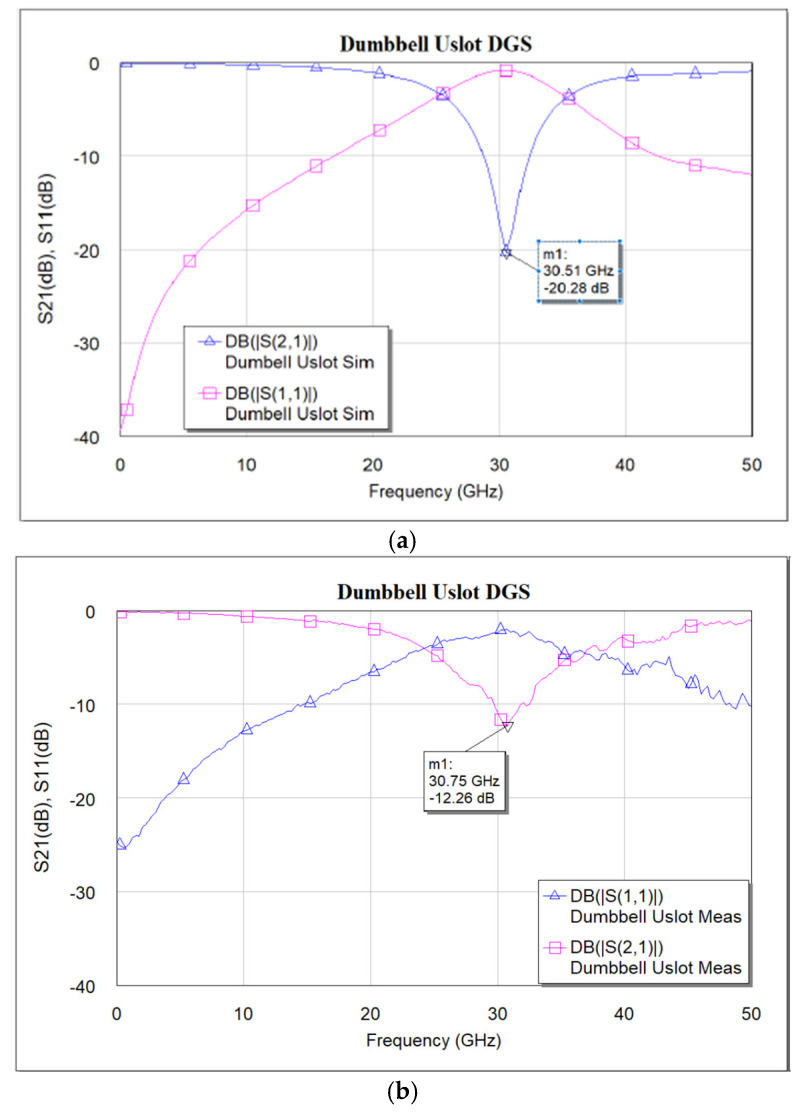

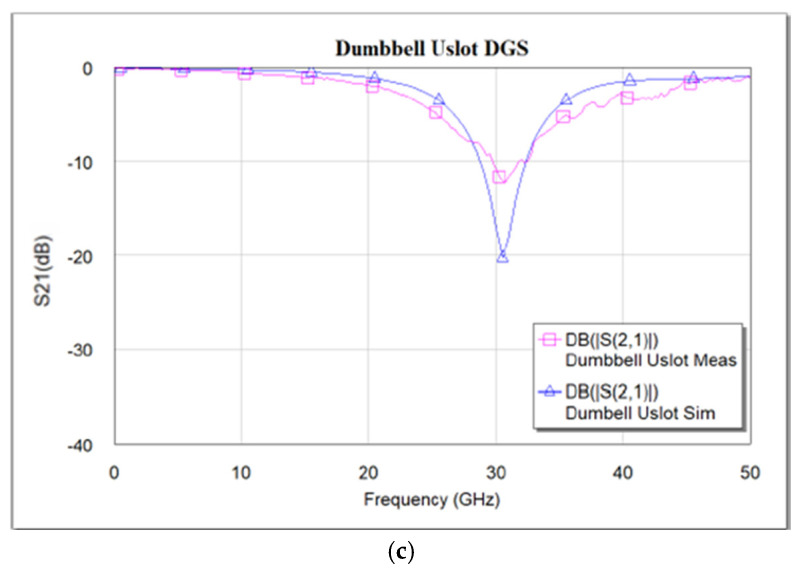
(**a**) Simulated frequency response of dumbbell u-slot DGS. (**b**) Measured frequency response of dumbbell u-slot DGS. (**c**) Simulated vs. measured S_21_ frequency response of dumbbell u-slot DGS.

**Figure 6:**
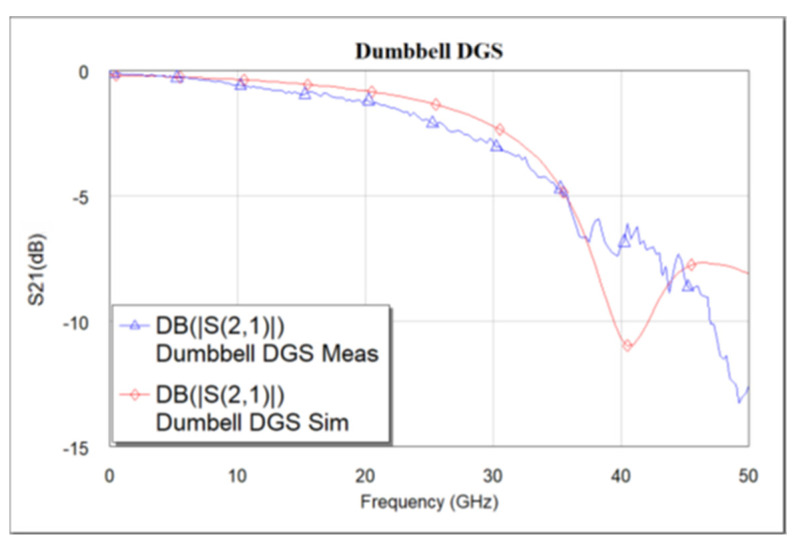
Simulated vs. measured S_21_ frequency response of dumbbell DGS.

**Figure 7:**
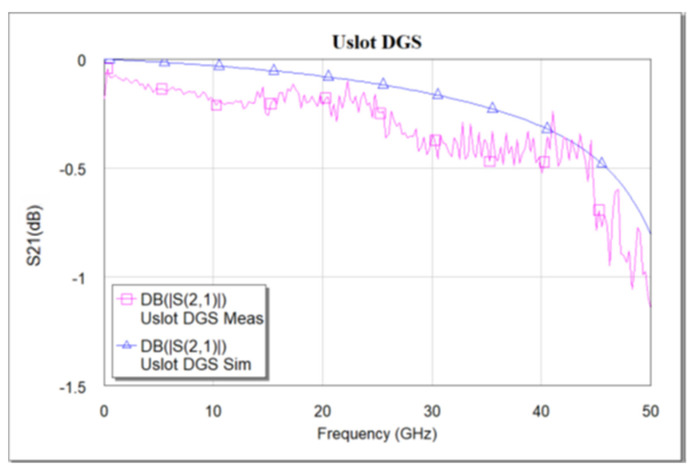
Simulated vs. measured S_21_ frequency response of u-slot DGS.

The designed devices have been analyzed using NI AWR design environment 15. BST ε_r_ = 700 is used for all simulations. As mentioned earlier, the CPW line is designed to have a characteristic impedance of 50 Ω. Figure 5a–c shows the simulated and measured frequency response of the dumbbell u-slot DGS shown in Figure 3. Figure 6 and Figure 7 show the simulated vs measured frequency response of the dumbbell DGS and u-slot DGS shown in Figure 1 and Figure 2, respectively. G-S-G probes with 150 µm pitch are used to measure the RF performance of the filter. The rectangular patch of the dumbbell DGS increases the series inductance, which in turn increases the reactance of the transmission line with an increase in frequency. This initiates the rejection of certain frequencies. The position of the attenuation pole is determined by the series inductance in parallel with the capacitance. This acts like a parallel LC resonator. As can be seen in Figure 6, the measured results show that the dumbbell DGS resonates around 50 GHz. Whereas from Figure 7, it is evident that the u-slot defect alone does not generate enough capacitance and inductance for the filter to resonate in the frequency of interest. To shift the resonance to a lower frequency, we must increase the inductance and capacitance, i.e., we must increase defect dimensions in each case. However, by combining both defects we can see resonance around 30 GHz for the same defect sizes; this helps in reducing the overall size of the filter. Having a high-dielectric material like BST also helps to reduce the size of the device, as we can get large capacitance and inductance values with small size defects.

The resonant frequency is given by the following equation:

(1) $$f=\frac{1}{2\pi \sqrt{lc}}$$

where *l* is the inductance and *c* is the capacitance of the device. A more detailed parametric analysis of these defects is presented in [15].

### Equivalent Circuit Model

**Figure 8:**
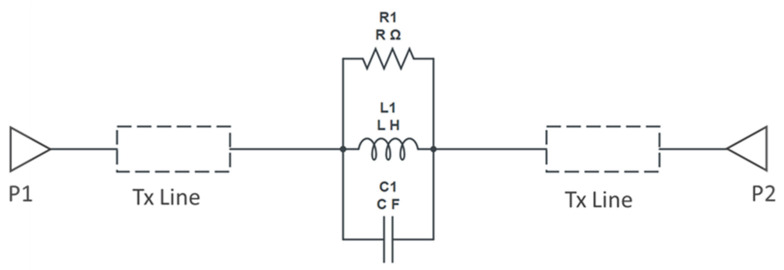
Circuit model for dumbbell u-slot DGS.

The designed device is also modeled using electrical equivalent circuits. The RF performance of the filter can be modeled using lumped elements. As mentioned earlier, the DGS section acts like an LC resonator; it can be modeled by a parallel LC resonator circuit [13]. However, to take the losses into account, it can be efficiently modeled using parallel RLC resonant circuits. The resistance is due to different losses such as conductor, dielectric and radiation losses. In [22], the equivalent circuit parameters *L*, *C* and *R* of the dumbbell shape were provided as follows:

(2) $$C=\frac{\;\;\omega_{c}}{2Z_{0}\left(\omega_{0}^{2}-\omega_{c}^{2}\right)}$$

(3) $$L=\frac{1}{\omega_{0}^{2}C}$$

(4) $$R\left(S11\left(\omega \right)\right)=\frac{2Z_{0}}{\sqrt{\frac{1}{{\left|S_{11}\left(\omega \right)\right|}^{2}}-{\left(2Z_{0}\left(\omega C-\frac{1}{\omega L}\right)\right)}^{2}}-1}$$

where $\omega_{0}$ is the angular resonance frequency, $\omega_{c}$ is the 3-dB cutoff angular frequency and $Z_{0}$ is the characteristic impedance of the transmission line. In [12,23], an improved equivalent circuit parameter extraction method for a dumbbell-shape DGS is proposed. However, for the dumbbell u-slot DGS proposed here, the equivalent circuit parameters are extracted using the AWR tool, which provides a variable tuner to fine-tune the parameters. The parameters are tuned to match the measured results. Figure 8 shows the equivalent circuit model used to extract circuit parameters for the dumbbell u-slot DGS. The two transmission line blocks used are for representing the transmission line before and after the defect, while the defect is represented by a parallel RLC circuit with R = 560 Ω, C = 0.136 pF and L = 0.198 nH for the dumbbell u-slot DGS.

## 4. Cascading of Dumbbell U-Slot DGS

**Figure 9:**
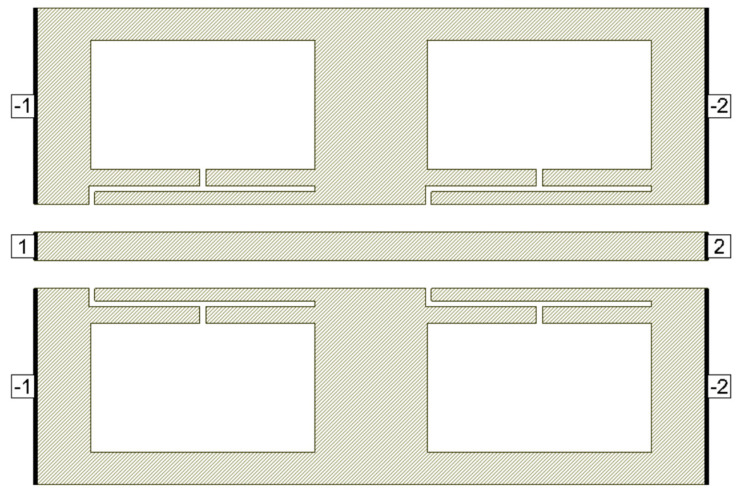
Dumbbell u-slot DGS cascade.

**Figure 10:**
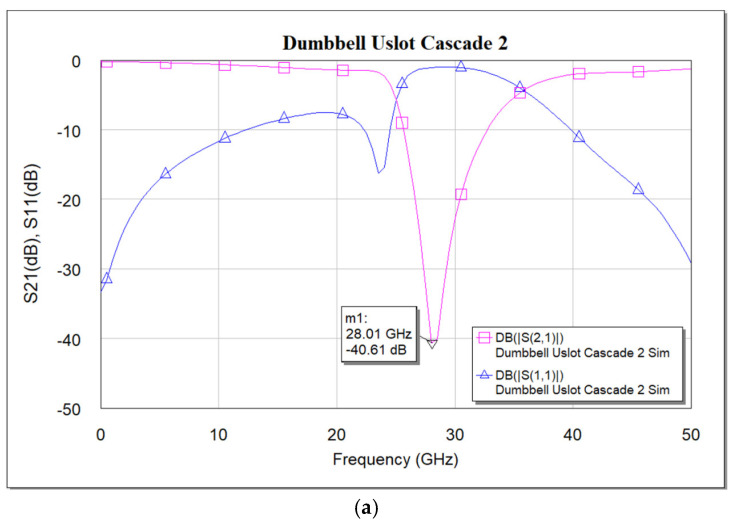

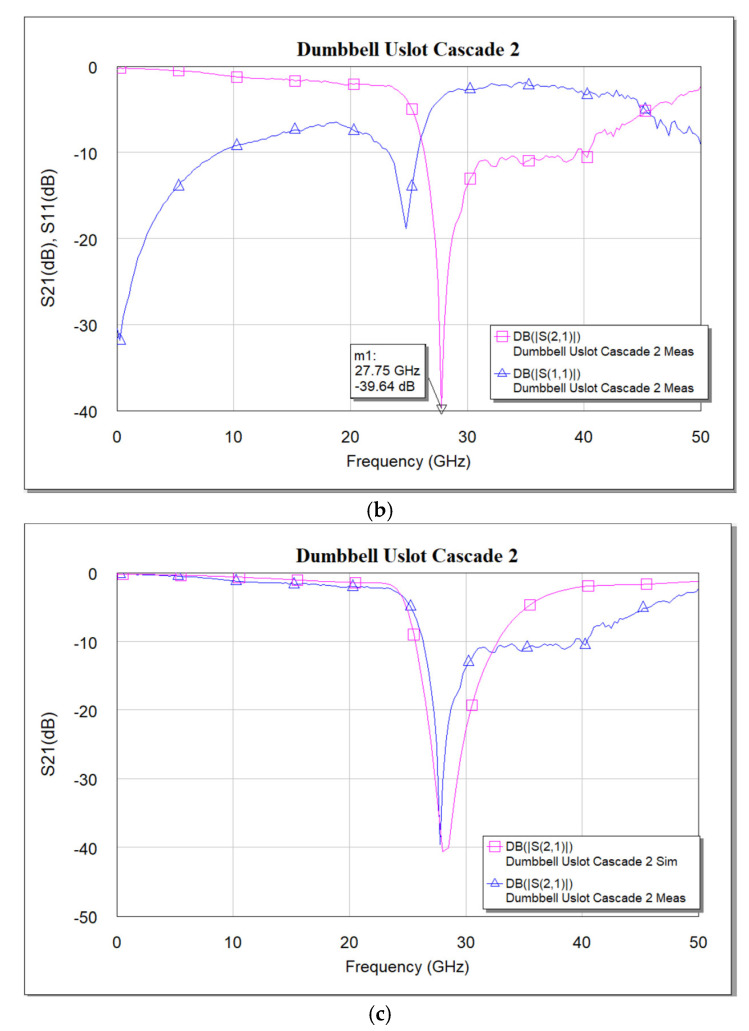
(**a**) Simulated frequency response of cascaded dumbbell u-slot DGS. (**b**) Measured frequency response of cascaded dumbbell u-slot DGS. (**c**) Simulated vs. measured S_21_ frequency response of cascaded dumbbell u-slot DGS.

Deeper rejection is achieved by cascading multiple standalone filter units. Here, a cascade of two dumbbell u-slot DGS filters is demonstrated; as we increase the number of cascades, deeper rejection is achieved and cascading occurs along the transmission line direction. Figure 9 shows the filter layout of cascading of two dumbbell u-slot DGSs. The same dimensions as shown in Figure 3 are used. Any change in the dimensions causes changes in the capacitance and inductance values, which in turn yield more than one resonance frequency. Variations in the film thickness can also yield multiple resonances in the frequency response. When the capacitance and inductance of both single units are the same, a single resonance with deeper rejection and narrow bandwidth is observed, as shown in Figure 10a–c, where simulation and measured results are presented. As can clearly be seen in Figure 10, the results indicate that cascading the two-unit cells gives about −39.64 dB rejection compared to about −12.26 dB rejection from a single cell.

**Figure 11:**
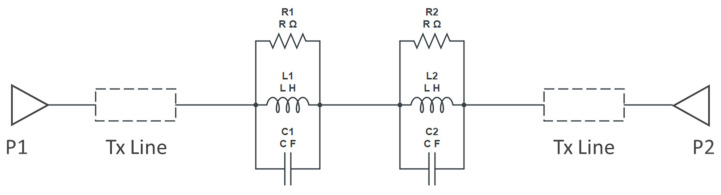
Circuit model for cascaded dumbbell u-slot DGS.

**Figure 12:**
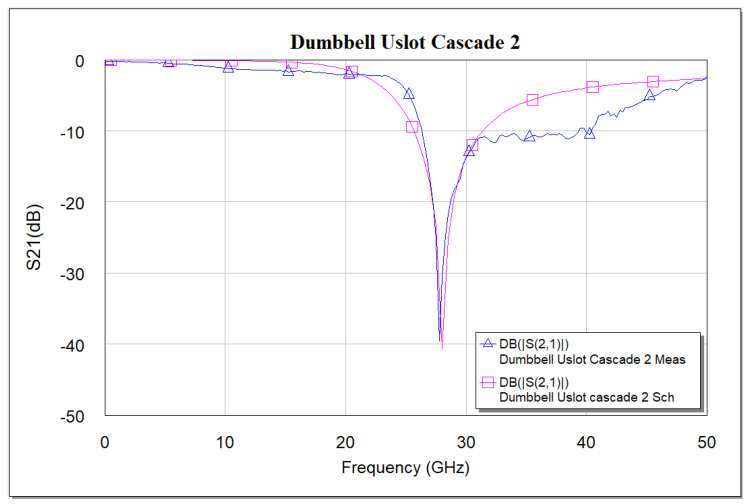
Schematic model vs. measured S_21_ frequency response of cascade dumbbell u-slot DGS.

The slight shift in the resonance frequency, as well as the narrow bandwidth from the measurements, are due to the combination of de-embedding errors and the CPW signal line’s parasitic coupling to the ground. For longer devices, the de-embedding errors play a vital role. The higher insertion loss after the notch is also mainly due to the above-mentioned reasons along with dielectric losses of the BST thin film. As a notch filter, the circuit behaves inductively, and after resonance it behaves capacitively. The modified varactor device with metal–insulator–metal capacitor (MIMCAP) structure using BST thin film typically has a Q-factor of about 5 with no DC bias voltage applied, and the Q increases to 8 with the application of 10 V DC bias voltage [24]. The equivalent circuit model is the same as shown in Figure 8, but cascaded twice to replicate the two cascaded DGS devices; the equivalent circuit model is shown in Figure 11, and the values extracted from the circuit diagram are R1 = R2 = 8650 Ω, C1 = C2 = 0.233 pF, L1 = L2 = 0.1382 nH. Increased resistor values show deeper rejection at the resonance frequency. The frequency response of the circuit model aligns very well with the measured data; the comparison is shown in Figure 12.

## 5. Tunable DGS Filter

**Figure 13:**
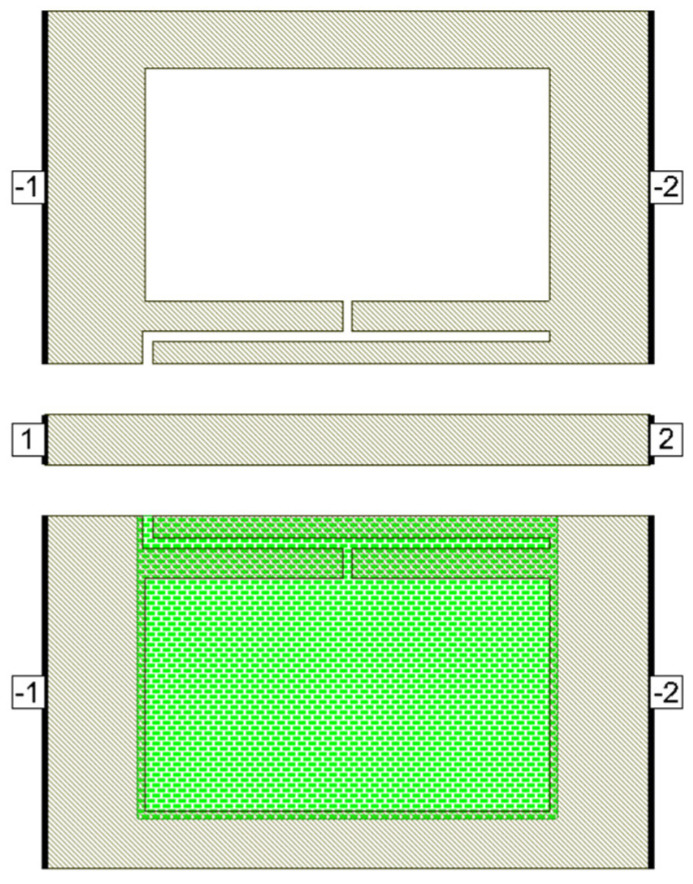
Dumbbell u-slot DGS on CPW line with PCM.

In general, tuning can be achieved by altering the resonator’s length or its inductive or capacitive loading, preserving its transmission and reflection properties throughout the given range of frequencies [25]. The BST material’s dielectric properties can be tuned by applying DC bias voltage; as the DC bias voltage is increased, the dielectric constant ε_r_ is reduced, which in turn reduces the capacitance of the device, making it a tunable element. However, achieving tuning with planar structures requires high DC bias voltages, unlike MIMCAP structures [24,26,27] where the BST material is sandwiched in between two metal layers which require lower DC bias voltages. In [28], a BST-based tunable surface acoustic wave (SAW) sensor is presented where the tunability is achieved by changing the capacitance of the sensor. For these planar DGS structures, DC biases from 0–21 V were applied but no tuning was observed. This is mainly because the smaller slot dimension in the device is 10 μm and the overall area of this slot compared to the device size is very small. Typically, 40 kV/cm is required to tune the devices. Highest tuning is achieved at 400 kV/cm. For this device, larger voltages of about 40 V or higher are required to see the tuning. However, in this case, having a BST thin film layer gives large capacitance and inductances which makes the size of the device small. Tuning is achieved by adding a phase change material (PCM) layer in the device, as shown in Figure 13. Here, a germanium telluride (Ge_0.5_Te_0.5_ (GeTe)) thin film layer is used as the PCM layer. GeTe is a chalcogenide phase change material which possesses a metal-to-insulator transition property. The as-deposited GeTe has an amorphous state, i.e., high resistance or insulator state. GeTe has a negative temperature coefficient of resistance, and it undergoes an insulator-to-metal or amorphous to crystalline phase at a transition temperature of 240 °C. The crystalline phase is a low-resistance phase. Once it reaches the low-resistance or crystalline state, it remains there. To return the material to its amorphous or high-resistance state, the material must be heated beyond its melting temperature for a very short time (in the order of a few nanoseconds); the atoms are then randomly distributed, making the material’s resistance high.

**Figure 14:**
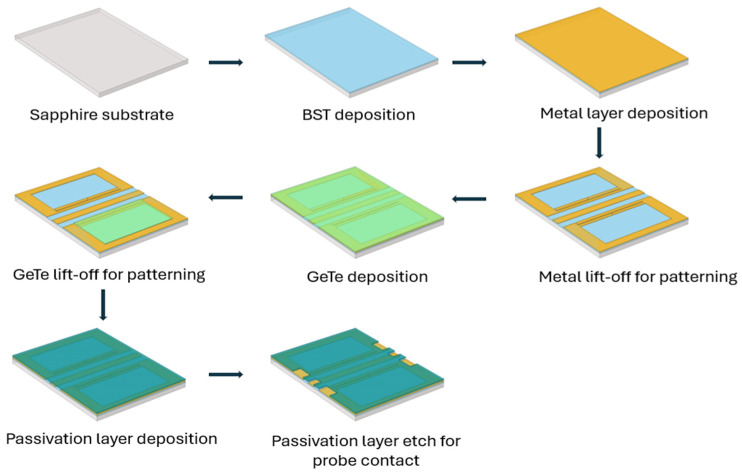
Schematic representation of the fabrication process of a dumbbell u-slot DGS using CPW line configuration with BST and GeTe thin films.

Figure 14 shows the schematic representation of the fabrication process of the DGS device with a PCM layer. The fabrication process is as shown in Figure 4, with only a few additional steps of GeTe deposition and patterning. The deposition process for GeTe is very simple: it is deposited at room temperature in a vacuum chamber with chamber pressure of about 5 × 10^−6^ Pa, using the PLD system as mentioned earlier. A pulse repetition frequency of 10 Hz is used with laser fluence adjusted to get about 3.6 J/cm^2^ at the GeTe target. GeTe thickness of 200 nm is deposited, and the lift-off process is used to pattern the GeTe. More details on GeTe film characterization are presented in [26].

**Figure 15:**
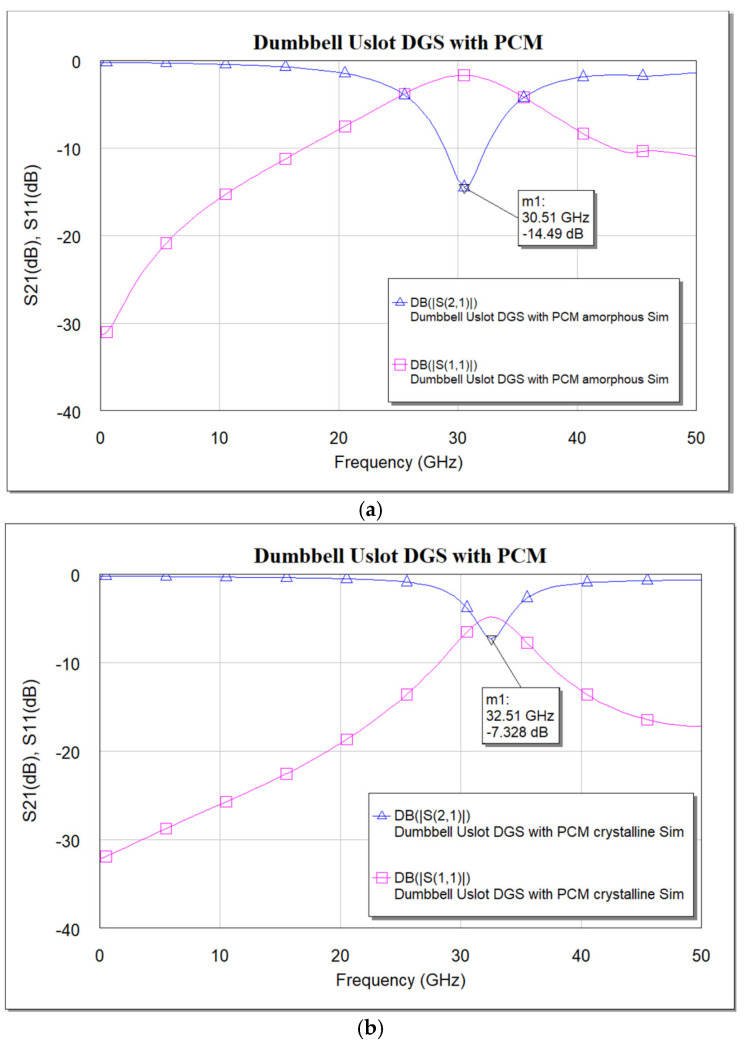

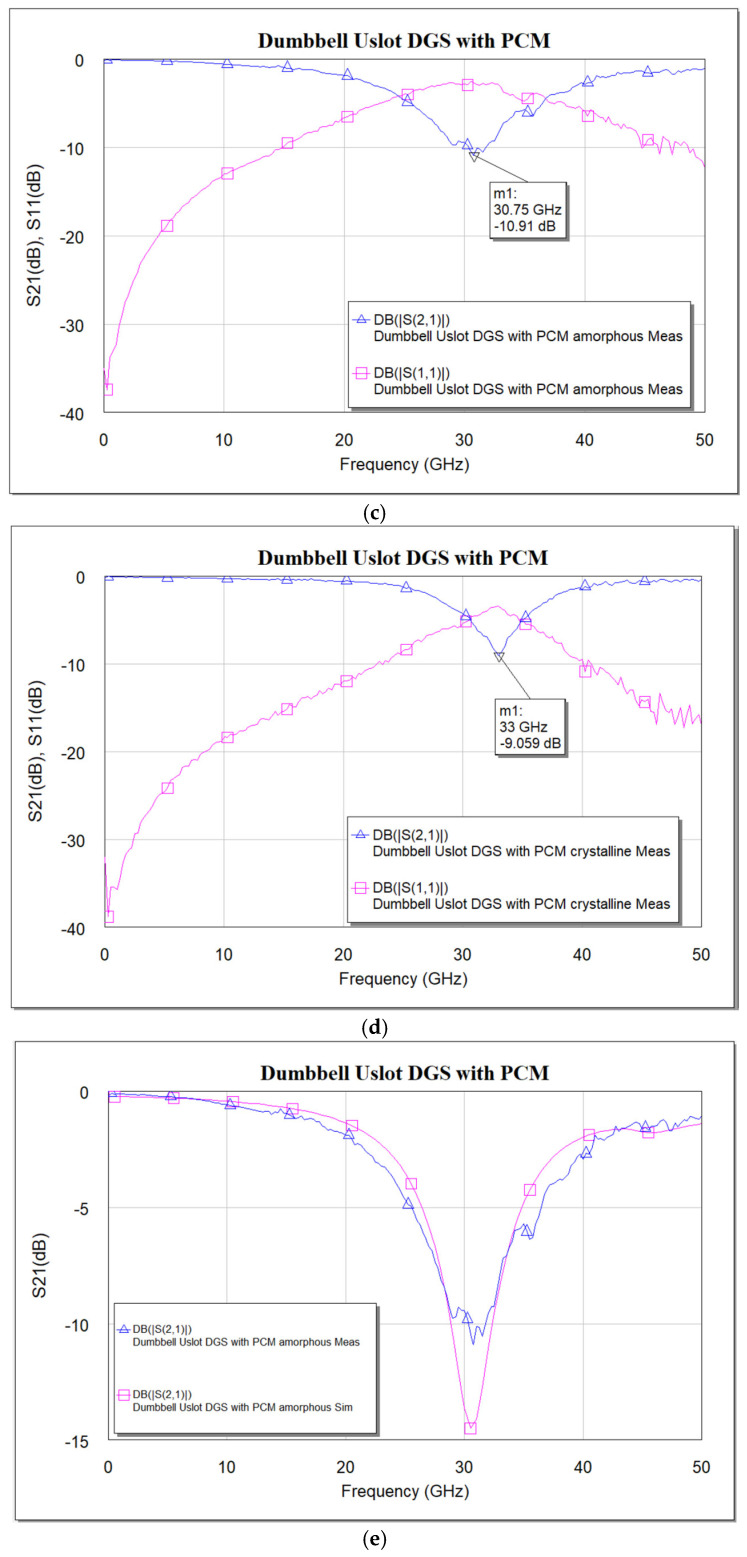

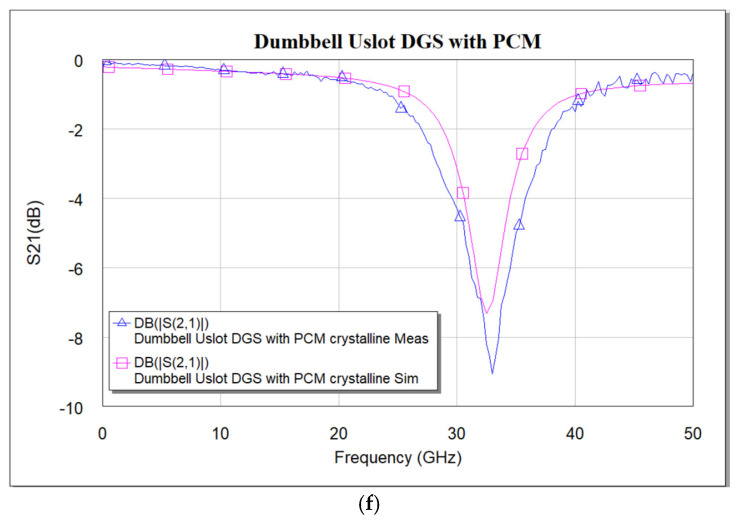
Frequency response of dumbbell u-slot DGS with PCM. (**a**) Simulation—amorphous state. (**b**) Simulation—crystalline state. (**c**) Measured—amorphous state. (**d**) Measured—crystalline state. (**e**) Simulation vs measured S_21_—amorphous state. (**f**) Simulation vs measured S_21_—crystalline state.

When the PCM is in the amorphous state, the device has inductance and capacitance from the defects in both the ground planes. But when the PCM is in a crystalline state, only inductance and capacitance from defects in one ground plane are seen, as the defects in the other ground plane would be covered with metal. This reduction in the inductance and capacitance will push the resonance to a higher frequency. This can be used for tuning the filter. Further, the tuning range can be adjusted with a PCM layer covering the defect, i.e., partial covering of the defect with PCM would adjust the tuning range accordingly. Figure 15 shows the simulated and measured frequency response of the dumbbell u-slot DGS in both amorphous and crystalline states.

**Figure 16:**
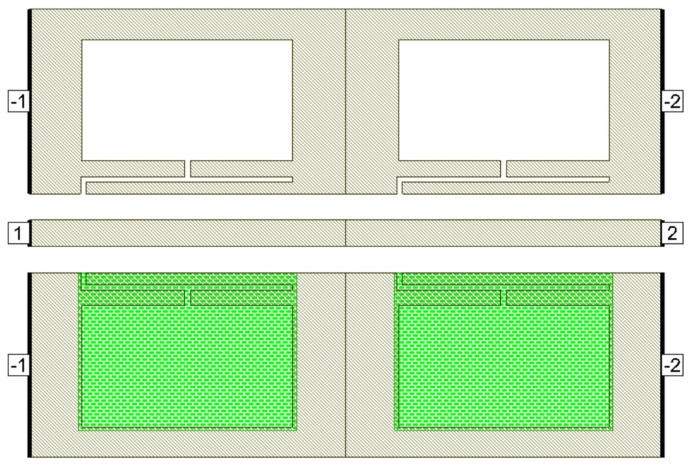
Dumbbell u-slot DGS with PCM cascade.

The frequency response in the amorphous state is like that of the dumbbell u-slot DGS: as the PCM is in the amorphous state, the device has inductance and capacitance from the defects in both the ground planes. However, we can see from the frequency response in the crystalline state that the resonance frequency has shifted to a higher frequency, as expected. From Figure 15c,d, it is clear that a shift in resonance frequency from 30.75 GHz to 33 GHz can be seen. A frequency shift of about 2.25 GHz is achieved, i.e., 7.32% of tuning is achieved by using the PCM. The measurements for the amorphous state are taken in the as-deposited state, where the PCM is in a high-resistance state. To make it crystalline, the sample is heated on a hotplate at 240 °C for about 5 min. The germanium telluride thin film transforms from amorphous to crystalline state. The resistivity of the germanium telluride is measured to confirm the crystalline state. GeTe tends to oxidize if heated in open atmospheric conditions; however, the presence of passivation prevents oxidation. Once the sample is cooled down, the measurements are again performed on the same device to see the change in the frequency response. Simulation vs measured results align well. The circuit model shown in Figure 8 is used to extract the parameters for both amorphous and crystalline states. For the amorphous state, R = 430 Ω, C = 0.159 pF, L = 0.171 nH and for the crystalline state, R = 430 Ω, C = 0.315 pF, L = 0.075 nH. The increase in capacitance in the crystalline state makes sense, as a big piece of metal PCM in the crystalline state applies a large parasitic capacitance to the substrate. The significance of the use of PCM can be seen when two-unit cells are cascaded, as shown in Figure 16. The repeatability is still under investigation, as GeTe requires about 725 °C for re-amorphization. This high temperature damages the crystal structure of the BST thin film which is deposited under it. Efforts are underway to find alternative material combinations with which repeatability can be achieved.

**Figure 17:**
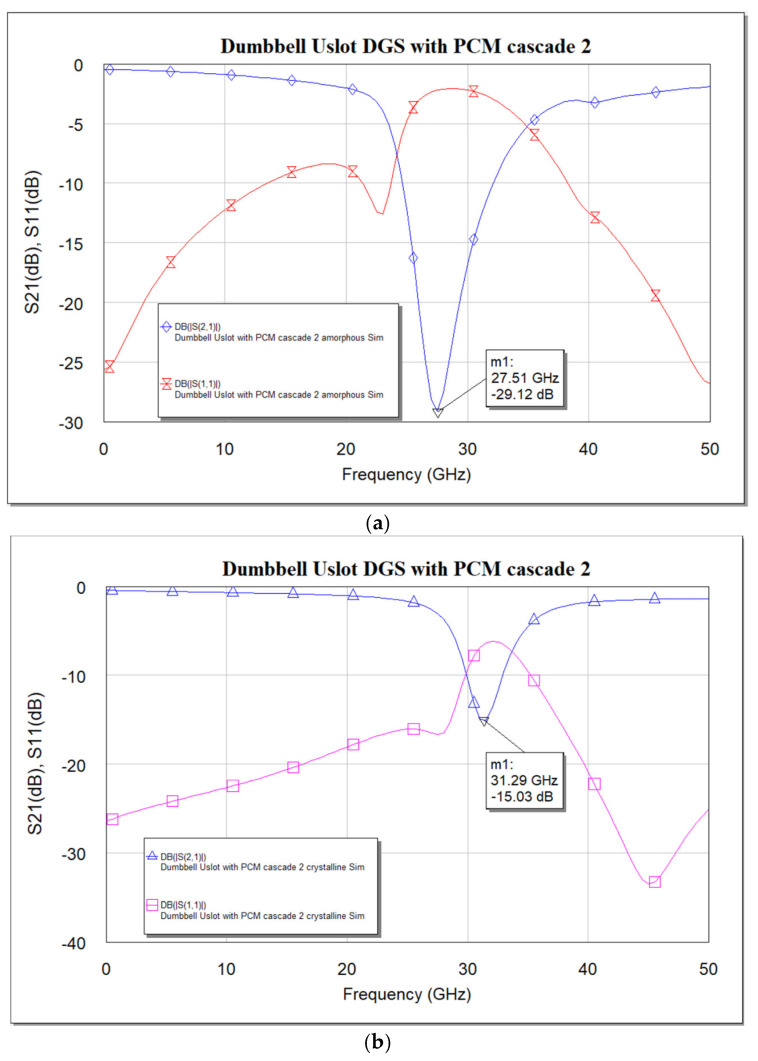

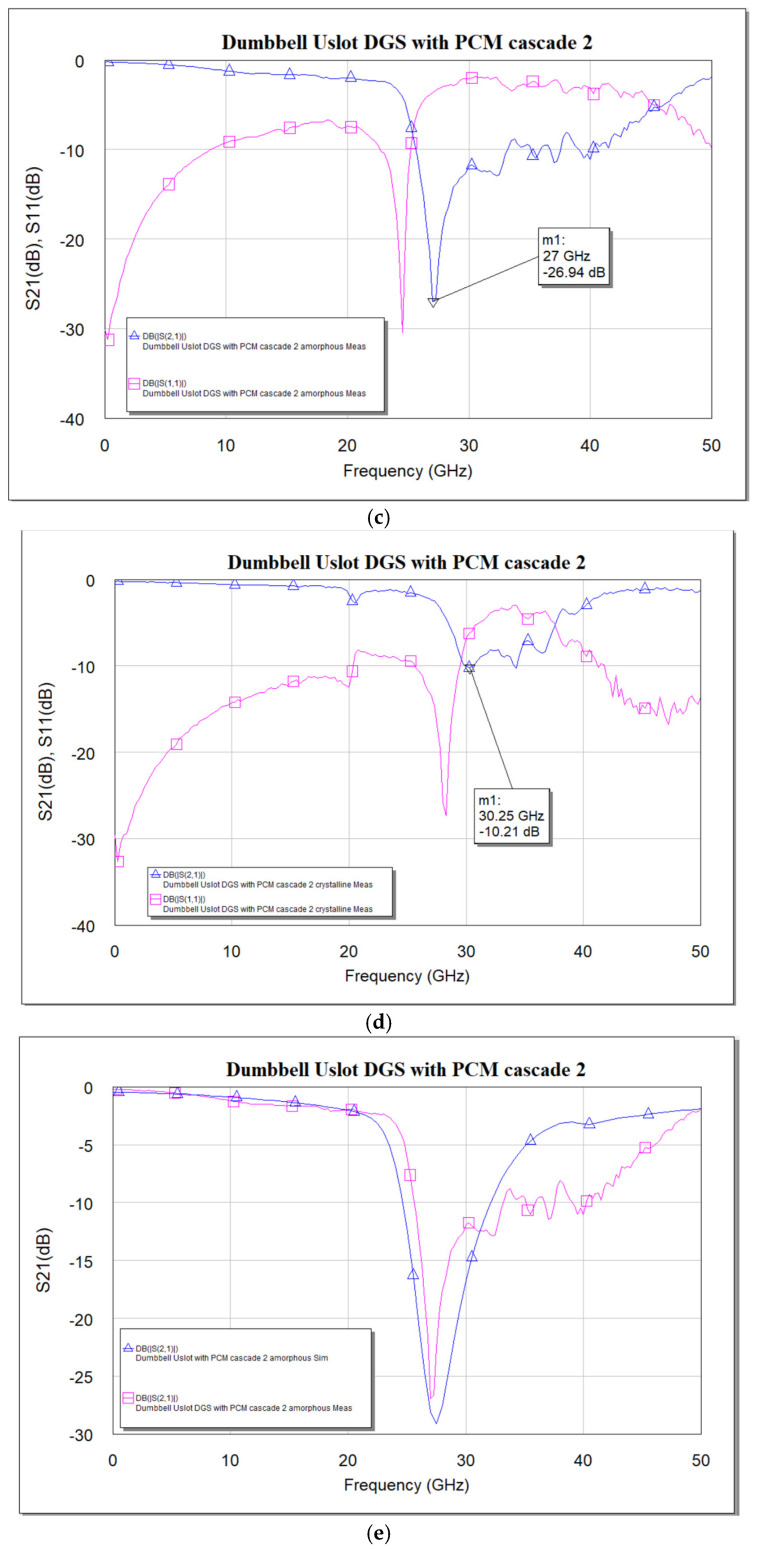

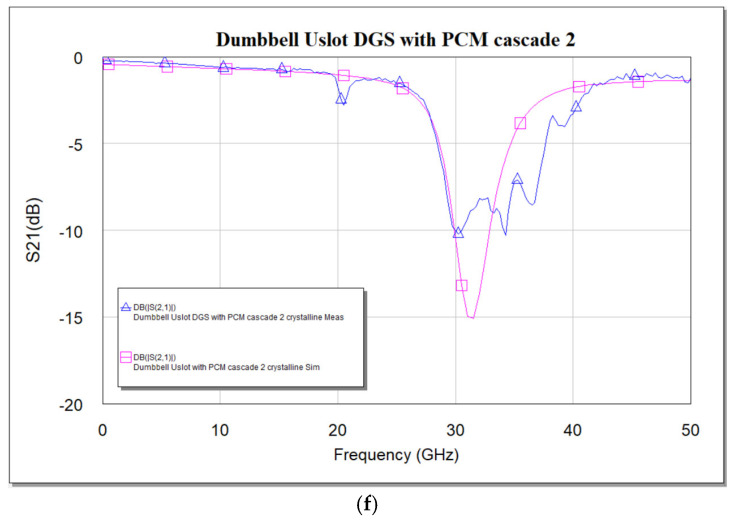
Frequency response of dumbbell u-slot DGS cascade with PCM. (**a**) Simulation—amorphous state. (**b**) Simulation—crystalline state. (**c**) Measured—amorphous state. (**d**) Measured—crystalline state. (**e**) Simulation vs measured—amorphous state. (**f**) Simulation vs measured—crystalline state.

Cascading clearly shows that the effect of using PCM is significant: from the measured data in Figure 17c,d, a shift in resonance frequency from 27 GHz to 30.25 GHz is seen. A frequency shift of about 3.25 GHz is achieved, i.e., 12.04% of tuning is achieved for a cascaded DGS with PCM compared to 2.25 GHz, i.e., 7.32% achieved using a single DGS cell with PCM. The simulation results align well with measurements.

## 6. Conclusions

In summary, a tunable defected ground structure using Ba_0.6_Sr_0.4_TiO_3_ and Ge_0.5_Te_0.5_ thin films has been successfully fabricated and measured. The potential of phase change materials (PCM) for dynamically tuning the frequency response of a dumbbell u-slot defected ground structure (DGS)-based band stop filter has been explored. Lower insertion loss of less than −2 dB below the lower cutoff frequency and enhanced band-rejection with notch depth of −39.64 dB at 27.75 GHz was achieved by cascading two-unit cells, compared to −12.26 dB rejection with a single-unit cell using BST thin film only. Tuning of the frequency response was achieved by simply adding a phase change material layer deposited on to the device with simple fabrication steps. About 7.32% tuning was achieved for dumbbell u-slot devices with PCM, and the tuning range was increased to 12.04% by cascading two-unit cells of DGS. Further cascading of more than two-unit cells could increase the tuning range, but with some tradeoffs. Simulation results align well with the measured results. The repeatability is still under investigation, as GeTe requires about 725 °C for re-amorphization. This high temperature damages the crystal structure of the BST thin film which is deposited under it. Efforts are underway to find alternative material combinations with which repeatable tunability can be achieved.

## Data Availability

Data are contained within the article.
